# Incidence and Predictors of Diabetic Nephropathy among Type 2 Diabetes Mellitus Patients, Southern Ethiopia

**DOI:** 10.1155/2024/6976870

**Published:** 2024-07-04

**Authors:** Fasika Merid, Firdawek Getahun, Habtamu Esubalew, Tamirat Gezahegn

**Affiliations:** ^1^ Department of Public Health Arba Minch College of Health Sciences, Arba Minch, Ethiopia; ^2^ Department of Public Health College of Medicine and Health Sciences Arba Minch University, Arba Minch, Ethiopia

## Abstract

**Background:**

Diabetic nephropathy is the most common cause of end-stage renal disease, and it brings high morbidity and mortality. Globally, the predominant rise in type II diabetes prevalence significantly increases the incidence of diabetic nephropathy. Therefore, timely diagnosis and prompt management of diabetic nephropathy and early identification of predictors are essential. Thus, this study aimed to determine the incidence and predictors of diabetic nephropathy among type II diabetes mellitus patients.

**Methods:**

A retrospective follow-up study was conducted among 532 type II diabetes patients who enrolled at Hawassa University Comprehensive Specialized Hospital from January 1, 2012, to December 31, 2021. A simple random sampling technique was used to select the study participants. The extracted data were entered into EpiData version 3.1 and analyzed by Stata version 14. A bivariate and multivariable Cox proportional hazard regression analysis was fitted to identify predictors of diabetic nephropathy. The Cox proportional hazards assumption was checked using the Schoenfeld residual test, and the goodness of fit of the model was checked using the Cox–Snell residual test. An adjusted hazard ratio with a 95% confidence interval and *P* values were used to identify statistically significant predictors.

**Results:**

The overall incidence rate of diabetic nephropathy was 2.71 cases (95% CI: 2.12, 3.47) per 1,000 person-months of observation. Age (AHR = 1.027; 95% CI = 1.005, 1.049), fasting blood sugar (AHR = 1.010; 95% CI = 1.007, 1.013), and systolic blood pressure (AHR = 1.050; 95% CI = 1.031,1.069) were significant positive predictors of diabetic nephropathy, whereas the duration of diabetes longer than five years (AHR = 0.20; 95% CI = 0.09, 0.44) was a protective predictor for the development of diabetic nephropathy.

**Conclusion:**

The incidence rate of diabetic nephropathy was high. Age, fasting blood sugar, systolic blood pressure, and duration of diabetes were found to be independent predictors of diabetic nephropathy. To overcome this public health problem, prompt and effective strategies should be designed based on identified predictors to prevent the development of diabetic nephropathy.

## 1. Introduction

Diabetes is a group of metabolic diseases characterized by hyperglycemia resulting from defects in insulin secretion, insulin action, or both [1]. Diabetes mellitus (DM) is estimated to affect 463 million people by the year 2019 [2] and 537 million by 2021 globally, and this accounts for 10.5% of the global adult population aged 20 to 79 years. It affects 24 million people in Africa, with 1.9 million (3.3%) living in Ethiopia. According to the International Diabetes Federation, the number of individuals with DM is estimated to rise to 783 million by the year 2045 worldwide. By 2045, the number of people with diabetes will have increased by 94% in low- and middle-income countries and by 129% in Africa [3]. The increasing prevalence is mainly due to nutrition transitions, rapid urbanization, and sedentary behaviors [4].

Diabetes is a major cause of kidney failure, blindness, heart attacks, stroke, nerve damage, and lower limb amputation that significantly impacts the quality of life of an individual [5, 6]. Type 2 diabetes mellitus (T2DM) is a chronic, noncommunicable disease, and its complications have contributed tremendously to the burden of mortality and disability worldwide [7]. It constitutes over 95% of people with diabetes, so the majority of people who develop diabetic nephropathy (DN) do so because of T2DM [5, 8]. Globally, the predominant rise in type II diabetes prevalence over the next two decades will significantly increase the incidence of DN [9]. Diabetic nephropathy (DN), or diabetic kidney disease, is “a syndrome characterized by the presence of pathological quantities of urine albumin excretion, diabetic glomerular lesions, and loss of the glomerular filtration rate (GFR) in diabetics” [10].

Diabetic nephropathy is one of the most feared microvascular complications of DM, and it is highly prevalent throughout the globe [11, 12]. It is a major healthcare challenge that affects 20% to 50% of people living with diabetes and 40% of T2DM patients [8, 12]. Diabetes has become one of the leading causes of end-stage renal disease (ESRD) worldwide as a result of the epidemic [8, 10]. Approximately 20% to 40% of type II diabetes patients with microalbuminuria progress to clinical nephropathy, of which nearly 20% progress to ESRD after developing overt nephropathy [10]. The majority of the patients with DN die from ESRD [13].

All-cause mortality among diabetic patients with DN is nearly 30 times higher compared to those of diabetic patients without DN [11]. This will have significant social and economic consequences, particularly in developing countries [9]. Diabetes-related complications relate to the direct cost of hospitalization and indirect costs, which are the most significant contributors to premature mortality, disability, and absenteeism. Early detection and improved management of complications of diabetes will have benefits not only for people living with diabetes but also for the wider health economy [2].

In 2019, worldwide DN among T2DM was responsible for 2.5 million incident cases, 129.56 million patients, 405,990 deaths, and 9.87 million disability-adjusted life years (DALY), which increased by 156.49%, 94.78%, 172.39%, and 141.73%, respectively. In Africa, it was associated with 194.97 thousand incident cases, 9.9 million prevalent cases, 42,680 deaths, and 1 million DALY, which increased by 56.42 thousand, 4 million, 18,910, and 455,170 from 1990, respectively. The increasing rate of DN is associated with age, sex, and anemia [14]. The pathogenesis for the development and progression of DN is multifactorial and complex, which is the result of metabolic disorders, hemodynamic abnormalities, and hormone synthesis [15, 16].

Prior studies revealed that the predictors for the development of DN in developed countries were age, sex, albuminuria, hypertension, duration of DM, myocardial infraction, dyslipidemia, SBP, smoking, waist circumference, poor glycemic control, previous retinopathy, and previous cardiovascular disease [17, 18], while in developing countries, age, sex, proteinuria, hypertension, HDL-C, body mass index, history of cardiovascular disease, poor glycemic control, type of antidiabetic medication, baseline GFR, coronary heart disease, anemia, duration of diabetes, SBP, and serum uric acid were the main predictors for the development of DN [19–26]. Management of modifiable risk factors may help to reduce DN incidence soon [27]. Through focusing on economic advancement, social development, and attention to the environment, all 17 sustainable development goals (SDGs) set out to address many of the structural factors that affect kidney health [28]. This means that each SDG has the potential to improve the health of the kidney and prevent kidney disease by improving the individual's and society's general health and well-being and by protecting the environment [29, 30]. For reaching the SDGs, targeting DN will be an important consideration [31].

Compared to developed countries, DN is more common among people who live with diabetes in Africa due to delayed diagnosis, limited screening and diagnostic resources, poor glycemic control, and other risk factors, and inadequate treatment at an early stage [32]. Therefore, timely diagnosis and prompt management of DN, and early identification of predictors are highly important to tackle this serious public health problem [33]. As a prerequisite for appropriate policy development and fair priority setting within every country, the local burden of diabetic nephropathy and its predictors and the local capacity to early identify and manage such conditions must be determined [30]. In developing countries, including Ethiopia, most epidemiological studies have been limited to the prevalence of diabetic nephropathy cross-sectionally, lacking the identification of predictor variables. A study on the incidence and predictors of diabetic nephropathy among T2DM may allow healthcare providers to reduce the incidence of diabetic nephropathy by targeting modifiable predictors to improve the extent of the consequences, particularly in resource-constrained countries like Ethiopia. However, the incidence and predictors of diabetic nephropathy among patients with T2DM are less often studied, and to assess this issue, no follow-up study has been conducted in the study setting. Therefore, this study aimed to determine the incidence and predictors of diabetic nephropathy among type II diabetes mellitus patients at Hawassa University Comprehensive Specialized Hospital.

## 2. Methods and Materials

### 2.1. Study Design, Setting, and Period

A retrospective follow-up study was conducted among patients newly diagnosed with T2DM at Hawassa University Comprehensive Specialized Hospital from January 1, 2012, to December 31, 2021. Hawassa University Comprehensive Specialized Hospital is located in Hawassa town, which is about 278 kilometers away from the capital, Addis Ababa. Public health facilities in the town are as follows: 1 Comprehensive Specialized Hospital, 2 General Hospitals, 1 Primary Hospital, and 7 Health Centers. Data were collected from April 14 to May 14, 2022.

### 2.2. Study Population

All type 2 diabetes mellitus patients who were attending the diabetic follow-up clinic at Hawassa University Comprehensive Specialized Hospital were a source population. All newly diagnosed T2DM patients who were enrolled from January 1, 2012, to December 31, 2021, at Hawassa University Comprehensive Specialized Hospital were a study population. Patients aged greater than or equal to 18 years who were diagnosed with T2DM and were on diabetic follow-up at Hawassa University Comprehensive Specialized Hospital from January 1, 2012, to December 31, 2021, were included in this study. Patients with T2DM who had diabetic nephropathy at the time of diagnosis, those with an unknown date of diagnosis, and patients without medical records were excluded from the study.

### 2.3. Sample Size Determination and Sampling Technique

The sample size was determined using Stata software power analysis for the Cox proportional hazards model by considering the following assumptions: probability of type I error (*α*) 0.05, power 80%, variability of covariates of interest 0.5, adjusted hazard ratio (AHR) 1.74 from the previous study [25], probability of event 0.1965, and proportion of withdrawals 0.1. Finally, the required sample size was 579. Study participants who fulfilled the inclusion criteria were selected by simple random sampling techniques using computer-generated random numbers from the sampling frame.

### 2.4. Data Collection Procedure or Tool

The data extraction checklist tool was adapted after reviewing different literature [18–20, 25, 26] that were used to extract relevant data from the patient's medical record. The contents of the checklist were validated by expert seniors in the field. The checklist includes sociodemographic characteristics and clinical and treatment-related factors. The data were collected by three BSc nurses under the supervision of one public health professional. The data were collected by reviewing the T2DM patient's medical record.

### 2.5. Data Quality Assurance

A pretest using 5% of the sample size was conducted on the same setting before the actual data collection, and adjustment was made to the data extraction checklist based on the result of the pretest. One-day training was given on the objectives of the study and how to retrieve records as per the data extraction sheet to data collectors and supervisor before the actual data collection. The extracted data were cross-checked with random samples for consistency and completeness. During data collection, the supervisor and the principal investigator checked the extracted data for its completeness and consistency.

### 2.6. Study Variable

The dependent variable was the incidence of diabetic nephropathy, and the independent variables were sociodemographic variables (age, sex, and residence), clinical, and treatment-related variables (duration of diabetes, fasting blood sugar (FBS), systolic blood pressure (SBP), hypertension, diastolic blood pressure (DBP), family history of diabetes, triglyceride, cholesterol, low-density lipoprotein (LDL), high-density lipoprotein (HDL), proteinuria, retinopathy, neuropathy, cardiovascular disease (CVD), anemia, and type of antidiabetic treatment).

### 2.7. Operational Definitions

Event: Development of diabetic nephropathy within the follow-up period.

Censored: Patients who did not develop DN until the end of the study, or died, or lost to follow-up or transfer out before developing DN within the study period.

Diabetic nephropathy, defined as eGFR <60 ml/min/1.73 m^2^ estimated by the Cockcroft-Gault equation, present for greater than three months, and/or persistent albuminuria [8, 34].

Hypertension is defined as an average SBP ≥140 mmHg or DBP ≥90 mmHg or both that were confirmed on two separate visits or are on prescription medication for hypertension [35, 36].

### 2.8. Data Processing and Analysis

The collected data were checked for completeness and consistency, and then entered, coded, edited, and cleaned using EpiData version 3.1 and exported to Stata version 14 for further analysis. The incidence rate of DN was calculated by dividing the number of people who experienced DN during the follow-up period by the person-time at risk starting from the time of T2DM diagnosis until the end of each patient follow-up period. Descriptive statistical analysis, including frequencies with percentage, mean with standard deviation (SD), and median with interquartile range (IQR) was performed. A life table was used to estimate the cumulative probability of developing DN in T2DM at different time intervals. The Kaplan–Meier survival curve, together with the log-rank test, was used to compare the survival differences between different categories of independent variables. A Cox regression model was used to examine the relationship between the rate of DN occurrence and the influence of the predictor variables. Bivariate and multivariable Cox proportional hazard regression analysis were performed between dependent and independent variables to identify significant predictors of DN. Variables in bivariate analysis with a *p* value <0.25 were selected as candidate for multivariable Cox proportional hazard regression analysis to control the effect of confounders. The model was built using the backward stepwise likelihood ratio method. Variables with a *P* value <0.05 in the multivariable analysis were considered statistically significant. The assumption of Cox proportional hazard was checked using the Schoenfeld residuals test (Prob > chi^2^ 0.4085). The goodness of model fitness was checked by using the Cox–Snell residual. Multicolinearity between variables was checked using the variance inflation factor (mean VIF 1.07). An adjusted hazard ratio with a 95% confidence interval was used to show the presence and strength of the association, and *P* values were used to identify statistically significant predictors. Finally, the findings of the study were presented in text, tables, and figures.

### 2.9. Ethics Approval Statement

An ethical clearance letter was obtained from the institutional review board of Arba Minch University with reference number IRB/1242/3022. Then, an official support letter was obtained from the Department of Public Health, and similarly, permission was secured from Hawassa University Comprehensive Specialized Hospital. Confidentiality was maintained by assigning a code rather than personal identifiers. Due to the retrospective nature of the study, the requirement of informed consent was waived for reviewing the patient's medical records.

## 3. Results

### 3.1. Sociodemographic Characteristics

A total of 532 (91.9%) type II diabetes mellitus patients were included in the analysis with the exclusion of incomplete charts from the total sample size of 579 during the follow-up period. The mean age of the study participants was 49.4 years, with a standard deviation (SD) of ±12.5 years. More than half 292 (54.89%) of the study participants were male. Among the participants, three-fourths (74.81%) were urban residents (Table 1).

### 3.2. Clinical and Treatment Characteristics of Study Participants

The study participants fasting blood sugar and systolic blood pressure median with interquartile range (IQR) was 171 (83.5–258.5) and 125 (112–138), respectively. More than one-third (44.17%) of the participants were hypertensive. Out of the total study participants, more than three out of ten, 193 (36.28%), had a family history of diabetes mellitus. Thirty-three (6.2%) of the study participants had diabetic retinopathy, and seventy-nine (14.85%) had diabetic neuropathy. The total cholesterol level of one-third, 185 (34.77%), of the study participants' was greater than or equal to 200 mg/dl, and nearly one-fourth, 144 (27.07%), of their HDL was less than 40 mg/dl. More than half (57.33%) of the participants used oral hypoglycemic agents (OHA) (Table 2).

### 3.3. Incidence of Diabetic Nephropathy

**Figure 1:**
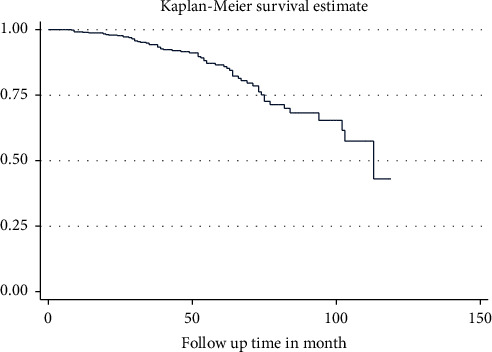
The overall Kaplan–Meier survival curve of type II diabetes mellitus patients at Hawassa University Comprehensive Specialized Hospital, Southern Ethiopia, 2012–2022.

**Figure 2:**
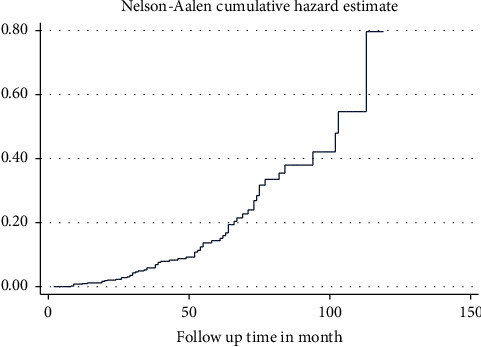
The Nelson–Aalen cumulative hazard estimate of type II diabetes mellitus patients at Hawassa University Comprehensive Specialized Hospital, Southern Ethiopia, 2012–2022.

With a total observation of 23,585, the overall incidence rate of diabetic nephropathy was 2.71 cases (95% CI: 2.12, 3.47) per 1,000 person-months of observation, or 32.52 cases (95% CI: 25.44, 41.64) per 1,000 person-years of observation. During the follow-up period, 64 (12.03%; 95% CI: 9.26, 14.80) developed diabetic nephropathy, and the median survival time to develop diabetic nephropathy was 113 months (Figures 1 and 2). The cumulative probability of survival was 99.2% in 12 months, 86.2% in 60 months, 69.1% in 84 months, and 41.8% in 120 months (Table 3).

### 3.4. Comparison of Survival Probability among Categories

**Figure 3:**
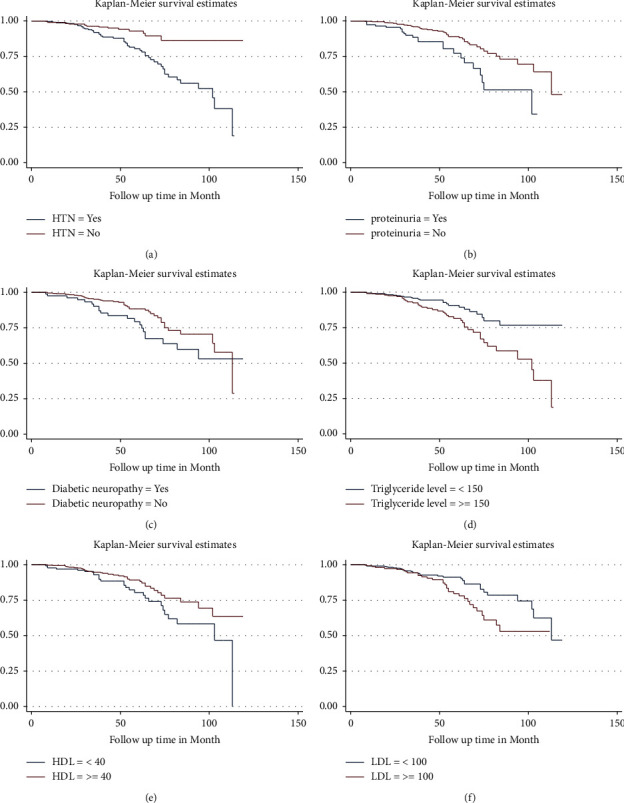
Kaplan–Meier survival curve showing the survival probability of developing DN by: (a) hypertension, (b) proteinuria, (c) diabetic neuropathy, (d) triglyceride level, (e) HDL level, and (f) LDL level among T2DM patients attending Hawassa University Comprehensive Specialized Hospital, Southern Ethiopia, 2022.

The Kaplan–Meier survival curve, together with the log-rank test, was used to compare the survival differences among various categories of predictor variables. Type II DM patients who had hypertension had a statistically significant lower survival probability compared to those who had no hypertension (*p* value 0.0001). Those who had a positive baseline proteinuria had a statistically significant lower survival probability compared to their counterparts (*p* value 0.0008). Type II DM patients with diabetic neuropathy had a statistically significant lower survival probability compared to those who didn't have diabetic neuropathy (*p* value 0.0397). Type II DM patients with triglyceride levels greater than or equal to 150 mg/dl had a statistically significant lower survival probability compared to those whose triglyceride level was less than 150 mg/dl (*p* value 0.0013). Those whose HDL level was greater than or equal to 40 mg/dl had a statistically significant higher survival probability compared to those whose HDL level was less than 40 mg/dl (*p* value 0.0289). In addition, T2DM patients whose LDL level was greater than or equal to 100 mg/dl had a statistically significant lower survival probability compared to those whose LDL level was less than 100 mg/dl (*p* value 0.0124) (Figure 3).

### 3.5. Predictors of Diabetic Nephropathy

In bivariate Cox proportional hazard regression analysis variables with *p* value <0.25, age, sex, residence, FBS, SBP, DBP, hypertension, proteinuria, diabetic neuropathy, cardiovascular disease, duration of diabetes, total cholesterol, triglyceride, HDL, and LDL were significantly associated with DN and were included in multivariable Cox proportional hazard regression analysis. However, age, fasting blood sugar, systolic blood pressure, and duration of diabetes were found to be independent predictors of diabetic nephropathy among T2DM patients.

For each one-year increase in age, the hazard of developing DN increased by 2.7% at any time during the follow-up period (AHR = 1.027, 95% CI = (1.005, 1.049)). As the fasting blood sugar level increases by 1 mg/dl, the hazard of developing DN increases by 1% at any time during the follow-up period (AHR = 1.010, 95% CI = (1.007, 1.013)). As systolic blood pressure increases by 1 mmHg, the hazard of developing DN increases by 5% at any time during the follow-up period (AHR = 1.050, 95% CI = (1.031, 1.069)). Among T2DM patients with a duration of diabetes longer than or equal to five years, the hazard of developing DN decreased by 80% when compared to their counterpart at any time during the follow-up period (AHR = 0.20, 95% CI = (0.09, 0.44)) (Table 4).

## 4. Discussion

The main goal of this study was to determine the incidence and predictors of diabetic nephropathy among T2DM patients. According to this study, the incidence rate of diabetic nephropathy among T2DM patients was 2.71 cases per 1,000 person-month observation. Age, fasting blood sugar, systolic blood pressure, and duration of diabetes were identified as independent predictors of DN.

The finding of this study revealed that the incidence rate of diabetic nephropathy was 2.71 per 1,000 person-month observation. The finding was consistent with a study conducted in St. Paul's Hospital, Addis Ababa, Ethiopia, 21.78 per 10,000 person month of observation [19], Black Lion, Addis Ababa, Ethiopia 3.6 per 100 person year of observation [25], and Southwest Ethiopia, 2.29 per 1,000 person month of observation [22]. This consistency might be due to the similarity in diagnostic methods, service provision, and follow-up period. However, the finding was slightly higher than the finding from the study conducted in Spain, which reported an incidence rate of 2.07 per 1,000 person-month of observation [18]. This discrepancy might be due to the differences in health care systems and the follow-up period of the study; in this study, 10-year data were used, whereas in a study conducted in Spain, 5-year data were used. The finding of this study was higher than the findings of the studies conducted in the Netherlands, 1,213 per 100,000 person year of observation [37]; Northwest Ethiopia, 14 per 10,000 person month of observation [20]; and Amhara region, Ethiopia, 193 per 10,000 person year of observation [26]. This difference could be related to the study setting and follow-up period [20, 26, 37]. In addition, for the study conducted in the Netherlands, the difference might be due to the sample size and variation in diagnostic methods [37]. Moreover, the black race had a higher incidence and severity of DN associated with a greater rate of GFR decline [27]. The incidence rate of DN was lower than in a study conducted in Iran, 43.84 per 1,000 person year of observation and 55.80 per 1,000 person year of observation [21]. These differences may be attributed to differences in urbanization, poor adherence to treatment, lifestyle, and methods used for defining the outcome [21]. Moreover, the possible explanation for the lower incidence rate of DN in our study may be due to the improvement of healthcare services nowadays compared to the previous.

In this study, age was a significant predictor of the development of diabetic nephropathy. The finding is in line with a study conducted in the United Kingdom, Spain, Ethiopia, and Iran [17–19, 21]. In older age, declining GFR is increasingly prevalent among cases with DN [27]. Among patients with T2DM, low eGFR, was very high in patients older than 65 years [38]. Due to the co-existence of prevalently increasing diabetes mellitus with age, renal function may be compromised in the elderly [39]. The reduction in renal function may be linked to cardiovascular hemodynamics due to endothelial cell dysfunction and changes in vasoactive mediators, resulting in increased atherosclerosis, glomerulosclerosis, and hypertension [40]. For diabetes in elderly DN patients, blood glucose control would have a greater benefit [39].

According to the findings of this study, type II diabetes patients with higher FBS levels were at a higher hazard of developing DN. The study finding is consistent with cohort studies conducted in Ethiopia and Iran, respectively [21, 25]. These might be explained by poorly controlled high blood sugar associated with the development and progression of DN [8, 12, 33]. Another possible explanation is that high blood sugar increases the worsening of renal function by altering the antioxidant system, which leads to the increased formation of advanced glycation end products and polyol pathway activation [8, 12]. In patients with T2DM who achieved optimal glycemic control, the incidence of DN decreased [33]. Thus, the risk of the onset of DN and its progression can be reduced through intensive glycemic control [8, 12].

In the current study, high SBP showed a significant association with DN. This is consistent with previous studies conducted in the United Kingdom, Asia, and Spain, respectively [17, 18, 23]. These might be due to the fact that higher blood pressure is a risk factor for both reduced GFR and microalbuminuria [27]. Controlling blood pressure is fundamental to reducing the risk of progression of DN [8]. For every 10 mmHg decrease in SBP, the development of DN is reduced by 17% [41]. Therefore, to slow the progression of DN, the most important intervention in patients with type II diabetes is a sustained reduction of blood pressure [42, 43].

Furthermore, the duration of diabetes mellitus is also the other predictor for the development of DN among T2DM patients. In this study, the duration of diabetes was negatively associated with the hazard of DN. The finding is supported by a study conducted in northwest Ethiopia [20]. However, the finding of the current study is inconsistent with those of studies done in Spain and Ethiopia, respectively [18, 25]. Patients with diabetes for a longer duration are more likely to develop DN than patients with a shorter duration of diabetes [44]. However, this discrepancy might be due to the study participants being T2DM patients; they may have been asymptomatic for several years and come to the health facilities lately when the diagnosis was finally made DN might have already been present [10, 44].

In the interpretation of the findings of this study, several limitations should be considered. First, due to the retrospective follow-up nature of the study, potential confounding factors like sociodemographic and behavioral characteristics (occupational status, educational status, smoking status, and physical activity) were not included due to the unavailability of the records. Second, in this study, variables like body mass index and hemoglobin A1c were incomplete. Third, as this study was institution-based, the findings might not be generalizable to the total population. The strength of this study was that patients with type 2 diabetes were followed for a longer duration, the sample size of this study was sufficiently powered, and the data were collected by trained health professionals.

This result suggests that in older age type 2 diabetic patients, diabetic nephropathy development and progression increase as the previous similar studies [17–19, 21]. This finding suggests that poor glycemic control accelerates the development of the incidence of DN over time, which is comparable with previous studies [21, 25]. These results build on existing evidence that uncontrolled blood pressure increases the progress towards the development of DN [17, 18, 23]. These results suggest that a longer duration of T2DM reduces the risk of developing DN, similar to the previous study [20]. However, this contradicts the existing evidence that a longer duration increases the hazard of DN development [18, 25]. Therefore, the implication to the researchers was to study the association between the duration of T2DM and the incidence of DN.

This finding indicates that DN remains a public health problem that deserves full policy consideration from policymakers to address and improve type II diabetes patient outcomes. If the high incidence of DN continues, it will result in huge health-related, economic, and social consequences to people living with diabetes and the healthcare system.

## 5. Conclusion

The study revealed that a substantial number of T2DM patients developed DN, with a high incidence rate in the study setting. Older age, high systolic blood pressure, and fasting blood sugar level were the identified predictors of DN. Duration of diabetes longer than five years was a negatively associated predictor with the development of DN. Tight systolic blood pressure and optimal blood sugar control could reduce the development and progression of DN among T2DM patients. Therefore, regular monitoring and counseling on blood pressure and blood sugar control should be strengthened for type II diabetes mellitus patient attendants at diabetic follow-up clinic, particularly for the older age.

## Figures and Tables

**Table 1:** Sociodemographic characteristics of type II diabetes patients at Hawassa University Comprehensive Specialized Hospital, Southern Ethiopia, 2022 (*n* = 532).

Variable	Categories	Frequency	Percentage
Age	Mean (SD) 49.4 ± 12.5 years		

Sex	Male	292	54.89
	Female	240	45.11

Residence	Urban	398	74.81
	Rural	134	25.19

**Table 2:** Clinical and treatment characteristics of type II diabetes patients at Hawassa University Comprehensive Specialized Hospital, Southern Ethiopia, 2022 (*n* = 532).

Variable	Categories	Frequency	Percentage
Fasting blood sugar	Median (IQR) 171 (83.5–258.5)		

Systolic blood pressure	Median (IQR) 125 (112–138)		

Diastolic blood pressure	Mean (SD) 78.81 ± 10.09		

Hypertension	Yes	235	44.17
	No	297	55.83

Family history of DM	Yes	193	36.28
	No	339	63.72

Anemia	Yes	21	3.95
	No	511	96.05

Proteinuria	Yes	114	21.43
	No	418	78.57

Diabetic retinopathy	Yes	33	6.20
	No	499	93.80

Diabetic neuropathy	Yes	79	14.85
	No	453	85.15

Cardiovascular disease	Yes	55	10.34
	No	477	89.66

Duration of diabetes	<5 years	322	60.53
	≥5 years	210	39.47

Total cholesterol	<200 mg/dl	347	65.23
	≥200 mg/dl	185	34.77

Triglyceride	<150 mg/dl	313	58.83
	≥150 mg/dl	219	41.17

HDL	<40 mg/dl	144	27.07
	≥40 mg/dl	388	72.93

LDL	<100 mg/dl	360	67.67
	≥100 mg/dl	172	32.33

Type of treatment	OHA	305	57.33
	Insulin	174	32.71
	Both	53	9.96

DM, diabetes mellitus; HDL, high-density lipoprotein; IQR, interquartile range; LDL, low-density lipoprotein; OHA, oral hypoglycemic agent; SD, standard deviation.

**Table 3:** Life table of T2DM patients at Hawassa University Comprehensive Specialized Hospital, Southern Ethiopia, 2022.

Interval in month	Beginning total	Number of DN	Number of censored	Cumulative survival	Standard error	95% CI
0–12	532	4	37	0.9922	0.0039	0.9794, 0.9971
12–24	491	6	58	0.9793	0.0065	0.9619, 0.9888
24–36	427	14	110	0.9425	0.0115	0.9151, 0.9612
36–48	303	8	84	0.9136	0.0150	0.8789, 0.9387
48–60	211	10	68	0.8620	0.0213	0.8141, 0.8983
60–72	133	10	51	0.7818	0.0309	0.7137, 0.8355
72–84	72	7	24	0.6906	0.0424	0.5991, 0.7652
84–96	41	2	19	0.6467	0.0497	0.5401, 0.7346
96–108	20	2	11	0.5575	0.0726	0.4049, 0.6856
108–120	7	1	6	0.4181	0.1324	0.1692, 0.6519

**Table 4:** A bivariable and multivariable Cox proportional hazard regression analysis for predictors of time to diabetic nephropathy among T2DM patients at Hawassa University Comprehensive Specialized Hospital, Southern Ethiopia, 2022 (*n* = 532).

Variable	Survival status		CHR 95% CI	AHR 95% CI	*p* value
	DN	Censored			
Age			1.05 (1.03, 1.07)	1.027 (1.005, 1.049)	0.014
Sex					
Male	42	250	1.36 (0.81, 2.28)	1.17 (0.66, 2.07)	0.592
Female	22	218	1	1	
Residence					
Urban	52	346	1.48 (0.79, 2.78)	0.96 (0.49, 1.87)	0.899
Rural	12	122	1	1	
FBS			1.01 (1.008, 1.013)	1.010 (1.007, 1.013)	0.001^*∗*^
SBP			1.06 (1.048, 1.082)	1.050 (1.031, 1.069)	0.001^*∗*^
DBP			1.07 (1.039, 1.093)	1.02 (0.99, 1.05)	0.238
Hypertension					
Yes	48	187	3.03 (1.72, 5.36)	0.72 (0.34, 1.53)	0.390
No	16	281	1	1	
Proteinuria					
Yes	23	91	2.35 (1.41, 3.93)	1.22 (0.70, 2.12)	0.490
No	41	377	1	1	
Diabetic neuropathy					
Yes	21	58	1.73 (1.02, 2.94)	1.58 (0.92, 2.72)	0.098
No	43	410	1	1	
CVD					
Yes	11	44	1.62 (0.84, 3.13)	1.09 (0.53, 2.25)	0.807
No	53	424	1	1	
Duration of diabetes					
<5 years	19	303	1	1	
≥5 years	45	165	0.57 (0.28, 1.13)	0.20 (0.09, 0.44)	0.001^*∗*^
Total cholesterol					
<200 mg/dl	35	312	1	1	
≥200 mg/dl	29	156	1.45 (0.88, 2.37)	0.99 (0.56, 1.77)	0.977
Triglyceride					
<150 mg/dl	24	289	1	1	
≥150 mg/dl	40	179	2.24 (1.35, 3.72)	1.12 (0.62, 2.03)	0.712
HDL					
<40 mg/dl	27	117	1.73 (1.05, 2.84)	1.14 (0.68, 1.92)	0.618
≥40 mg/dl	37	351	1	1	
LDL					
<100 mg/dl	33	327	1	1	
≥100 mg/dl	31	141	1.86 (1.13, 3.06)	1.22 (0.71, 2.11)	0.472

1 = reference; ^*∗*^*p* value <0.001; AHR, adjusted hazard ratio; CI, confidence interval; CHR, crude hazard ratio; CVD, cardiovascular disease; DBP, diastolic blood pressure; FBS, fasting blood sugar; HDL, high-density lipoprotein; LDL, low-density lipoprotein; SBP, systolic blood pressure.

## Data Availability

All relevant data are presented in the manuscript. However, data can be available from the corresponding author upon reasonable request.

## Abbreviations

AHR: Adjusted hazard ratio
CVD: Cardiovascular disease
DBP: Diastolic blood pressure
DN: Diabetic nephropathy
ESRD: End-stage renal disease
GFR: Glomerular filtration rate
HDL-C: High-density lipoprotein-cholesterol
IQR: Inter quartile range
LDL: Low-density lipoprotein
OHA: Oral hypoglycemic agent
SBP: Systolic blood pressure
SD: Standard deviation
T2DM: Type 2 diabetes mellitus.
